# Human West Nile virus infection: a meta-analysis of recent global data (2019–24)

**DOI:** 10.7189/jogh.15.04278

**Published:** 2025-10-17

**Authors:** Ahmad Adebayo Irekeola, Ahmad A Alshehri

**Affiliations:** 1 Basic Sciences Unit, School of Dental Sciences, Health Campus, Universiti Sains Malaysia, Kubang Kerian, Kota Bharu, Kelantan, Malaysia; 2 Microbiology Unit, Department of Biological Sciences, College of Natural and Applied Sciences, Summit University Offa, Offa, Kwara, Nigeria; 3 Department of Clinical Laboratory Sciences, College of Applied Medical Sciences, Najran University, Najran, Saudi Arabia; 4 Health Research Center, Najran University, Najran, Saudi Arabia

## Abstract

**Background:**

West Nile virus (WNV), an arbovirus of significant public health concern, is widely distributed across various regions, especially in Africa, Asia, Europe, and North America. However, the current global prevalence is unknown. We aim to provide an update on the recent prevalence of human WNV infection.

**Methods:**

We performed a systematic review and meta-analysis that adhered to standard reporting guidelines to offer a comprehensive overview of the virus's prevalence worldwide. We systematically searched four electronic databases, extracted relevant data, and computed pooled prevalence estimates using the DerSimonian-Laird random-effects model. We conducted subgroup and meta-regression analyses by region and study population and assessed study quality with the Joanna Briggs Institute critical appraisal tool for prevalence studies.

**Results:**

The global pooled seroprevalence of human WNV, based on studies using confirmatory neutralisation assays, was estimated at 3.6% (95% confidence interval (CI) = 2.0, 6.5), with a notably higher rate in North America (18.8%; 95% CI = 14.4, 24.1), followed by Africa (4.8%; 95% CI = 1.7, 13.2). South America had the least seroprevalence (1.7%; 95% CI = 0.9, 3.4). Based on the specific participant population, seroprevalence rates were found to be 4.7% (95% CI = 1.7, 12.6) among blood donors, 3.2% (95% CI = 1.0, 10.4) among clinical patients, and 2.3% (95% CI = 1.2, 4.4) among high-risk individuals. The global pooled molecular prevalence was estimated to be 0.1% (95% CI = 0.00, 0.02).

**Conclusions:**

While the global WNV prevalence rates are relatively low, targeted interventions are needed. These findings underscore the urgency of enhancing blood donor screening in high-seroprevalence regions, integrating advanced diagnostics (*e.g.* nucleic acid tests) into surveillance systems, and harmonising global reporting protocols to mitigate cross-border transmission risks.

**Registration:**

PROSPERO: CRD42024555398.

Emerging and re-emerging infectious diseases pose major threats to global health systems, economies, and societies [1,2]. Vector-borne diseases consistently demonstrate their ability to utilise ecological and biological systems, leading to significant illness and death worldwide [3]. Many diseases have complex transmission cycles, making prevention and control difficult. West Nile virus (WNV), a member of the *Flaviviridae* family and a well-known arbovirus, is a prime example [4]. It demonstrates the complex interplay of ecological, biological, and environmental factors that affect its transmission and geographic spread [5,6]. Confined initially to Africa after its discovery in Uganda in 1937, WNV has become a global public health concern [7,8]. The virus has spread with increasing frequency and severity to new locations and populations, demonstrating its adaptability [9]. Factors such as urbanisation and climate change have worsened the situation, increasing human vulnerability to the virus and sustaining its impact on public health [10,11].

The WNV is primarily spread through the bites of infected mosquitoes, especially those of the *Culex* genus, which are found in both cities and rural areas [12]. Birds are the primary reservoir hosts, maintaining the virus in natural transmission cycles. Humans and other animals are incidental, dead-end hosts, meaning they are unable to spread the virus to others [13]. The infection can manifest with a range of symptoms, from mild to severe disease [14]. However, in most cases, infections remain asymptomatic [14]. In severe cases, the virus can invade the central nervous system, potentially leading to severe neurological conditions like encephalitis, meningitis, or acute flaccid paralysis. Although these severe forms are uncommon, they carry a high risk of death and long-term health problems, particularly for the elderly and those with weakened immune systems or chronic diseases [14].

Therefore, understanding the status of human WNV infections is crucial for assessing epidemiology and developing effective strategies to mitigate the spread and control the infection. Nonetheless, evaluating the global prevalence of WNV can be challenging due to several factors. Differences in national and regional surveillance systems lead to underreporting and inconsistent data collection. Diagnostic limitations, such as cross-reactivity with other flaviviruses, also make accurate WNV identification difficult [15,16]. The high number of asymptomatic infections further contributes to underestimating the true prevalence and obscures the full impact on public health [17].

We aim to compile and analyse global data on the prevalence of WNV in humans from 2019 to 2024, providing a comprehensive estimate broken down by geographical location, specific population, and detection method. To our knowledge, this is the first meta-analysis to examine human WNV infection on a global scale. The chosen timeframe reflects our aim to assess the most recent epidemiological trends and provide an up-to-date picture of the current global burden. Understanding the current prevalence of WNV is critical not only for managing the current disease burden but also for enhancing worldwide preparedness and response to future outbreaks. Accurate estimates of WNV prevalence allow health authorities to allocate resources more effectively, focussing on areas most impacted by the virus for monitoring, mosquito control, and patient care. The compilation of global prevalence data in this review serves to bridge critical knowledge gaps, support the development of targeted therapeutic strategies, and reinforce coordinated public health efforts for effective WNV surveillance and control.

## METHODS

### Search for relevant literature

We conducted a comprehensive preliminary search in PROSPERO using relevant keywords and phrases to identify ongoing studies and avoid duplication. Following this, we developed and registered a detailed study protocol on PROSPERO (ID: CRD42024555398). To ensure thorough coverage, we did a literature search across four widely recognised international electronic databases: Scopus, PubMed, Web of Science, and ScienceDirect. Throughout the study, we adhered to the PRISMA guidelines [18], ensuring the robustness and credibility of our synthesis. We aimed to identify scholarly articles documenting the occurrence of human WNV infection.

We employed a rigorous search strategy that spanned four major databases (File S1 in the **Online Supplementary Document**). Although the search keywords were in English, we did not apply any restrictions regarding the language or geographical region of the studies to ensure inclusivity. We translated the non-English records into English using the Google Translate tool to facilitate data extraction and inclusion. We conducted the initial search on 8 June 2024 and an updated and final search on 11 November 2024. Subsequently, we consolidated all search results from the databases into the Mendeley reference management software. At this stage, we systematically removed duplicate entries and screened the remaining unique records against the predetermined eligibility criteria.

### Eligibility assessment

We carefully designed and rigorously applied the inclusion and exclusion process for this review. We included recent studies (2019–24) assessing the prevalence of human WNV infection within defined populations. To qualify, the evaluated samples must have been collected on or after 2019. In cases where samples spanned older and recent years, we extracted only the data from recent years (2019 and later). Exclusion criteria comprised review articles, editorials, letters, case reports, conference proceedings, book chapters, and studies that did not focus on prevalence evaluation within a specific human population. We also excluded studies examining symptoms, morbidity, and mortality in pre-established WNV-positive populations or those reporting disease outbreaks. We removed articles that lacked accessible full texts or did not contain human WNV prevalence data. We also excluded experimental studies, including those based on animal models. Additionally, we removed duplicate or redundant articles to ensure the accuracy and reliability of the analysis.

### Data extraction

Following the established eligibility criteria, the two authors independently evaluated all titles, abstracts, and full-text records retrieved from the search databases. In cases of conflicting evaluations, discussions were held between the authors to reach a consensus. The selected articles underwent a comprehensive review of their full texts. We systematically extracted key information, including the first author’s name, publication year, study design, study location, method of WNV testing, number of WNV-positive samples, and sample size, and recorded it in a standardised data extraction sheet.

### Statistical analysis and quality appraisal

We estimated the pooled prevalence of WNV in the included studies using a random-effects model based on the DerSimonian-Laird method. To address potential cross-reactivity among flaviviruses, we only included studies that employed confirmatory serological testing using neutralisation assays to estimate global seroprevalence. When initial screening was performed using non-confirmatory methods such as enzyme-linked immunosorbent assay (ELISA), we analysed the samples that were later confirmed positive by neutralisation assays. In studies that conducted both molecular and serological tests, we considered data from each testing method separately and incorporated it appropriately into the relevant pooled estimates. We used OpenMetaAnalyst, version 12 (Brown University, Providence, Rhode Island, USA) and Comprehensive Meta-analysis, version 3 (Biostat, Inc., Englewood, New Jersey, USA) for the analysis. We conducted a leave-one-out sensitivity analysis to assess the robustness of the pooled estimate. Further, we evaluated publication bias using funnel plots and Egger’s test [19]. We assessed heterogeneity among studies using Cochran’s Q (Q) test and quantified it using *I*^2^ statistics, with thresholds of 25% representing low heterogeneity, 50% moderate heterogeneity, and 75% high heterogeneity [20,21]. We conducted a subgroup analysis to assess WNV prevalence and explore sources of heterogeneity across different geographical regions and study populations. To further investigate heterogeneity, we performed meta-regression using the method of moments. We defined statistical significance as a *P* < 0.05.

Both authors assessed the study quality using the Joanna Briggs Institute critical appraisal tool for prevalence studies [22] (File S2 in the **Online Supplementary Document**). We scored each criterion as ‘yes’ (*i.e.* 1) or ‘no’ (*i.e.* 0), resulting in a total quality score ranging from 0 to 9. We considered studies with ≥7 scores to have adequate methodological quality [23].

## RESULTS

### Literature search outcomes and selected studies

**Figure 1:**
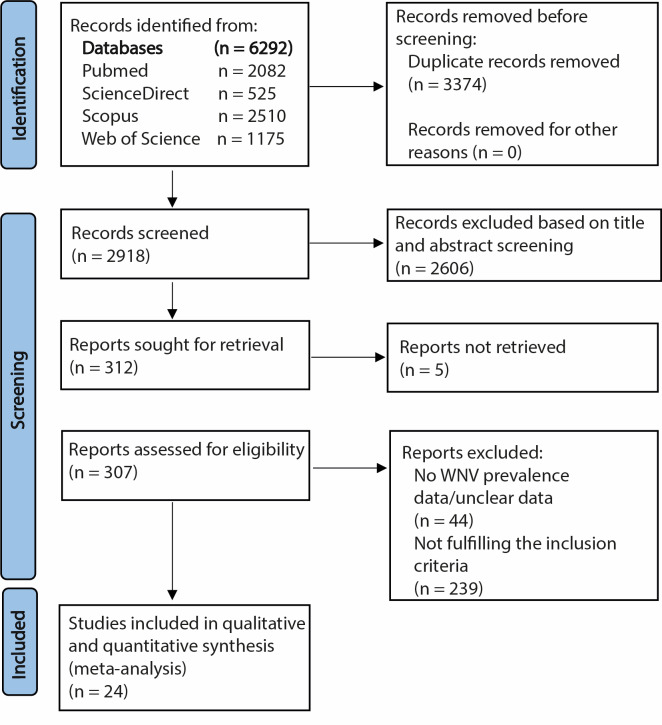
Summary of the literature search and selection for human WNV prevalence. WNV – West Nile virus.

During the literature identification phase, we retrieved 6292 records from an extensive search across PubMed, ScienceDirect, Scopus, and Web of Science. After eliminating 3374 duplicate entries, we screened 2918 records (**Figure 1**). In the screening stage, we evaluated 2918 records based on their titles and abstracts to identify pertinent studies that aligned with the research objectives and inclusion criteria. Consequently, we excluded 2606 records that did not meet the inclusion criteria. Lastly, we conducted a full-text review of the articles and ultimately identified 24 studies that were appropriate for both qualitative and quantitative synthesis.

### Characteristics of the included studies

We summarised the key characteristics of the 24 included studies, including geographical location, methodology, sample size, and study type (**Table 1**). These studies span multiple continents, highlighting the global nature of WNV. While some countries contributed only a single study, two studies from Romania, Russia, Senegal, Turkey, and the USA met the inclusion criteria. Most of the included studies employed cross-sectional designs. Further, the diagnostic methods used across the studies highlight the varied approaches to detecting WNV infection. Serological testing involving the ELISA predominated. Some studies used ELISA exclusively, while others conducted additional serological confirmation with neutralisation assays. Research from Brazil, the USA, Senegal, and Rwanda provided data on WNV prevalence using molecular techniques, including reverse transcription polymerase chain reaction and next-generation sequencing. Further, a quality assessment of the included studies demonstrated their overall high quality (File S3 in the **Online Supplementary Document**).

**Table 1:** Summary of the characteristics of studies on human WNV prevalence

Study, year, reference	Country	Study design	Method	Sample size, n
Alzuheir et al., 2021 [24]	Palestine	Cross-sectional	ELISA IgG	100
Assaid et al., 2021 [25]	Morocco	Cross-sectional	ELISA IgG, VNT	91
Bektore et al., 2024 [26]	Turkey	Cross-sectional	ELISA IgG, VNT	443
Blanco et al., 2024 [27]	Argentina	Retrospective	PRNT	400
Constant et al., 2022 [28]	France	NR	ELISA IgM/IgG, MNT	500
Coroian et al., 2022 [29]	Romania	Cross-sectional	ELISA IgG, SNT	1200
Crivei et al., 2024 [30]	Romania	Cross-sectional	ELISA IgG	88
de Bellegarde de Saint Lary et al., 2023 [31]	Netherlands	Cross-sectional	Protein microarray (IgG), FRNT	157
Kibathi et al., 2024 [32]	Kenya	Cross-sectional	PRNT	480
Kwon et al., 2023 [33]	USA	Retrospective	Procleix WNV Assay	51 867
Löwen Levy Chalhoub et al., 2021 [34]*	Brazil	Cross-sectional	PRNT, RT-PCR	78
MacIntyre et al., 2023 [35]	South Africa	Cohort	ELISA IgM, VNT	441
Marsland et al., 2024 [36]	Australia	Cross-sectional	ELISA	761
Nagy et al., 2022 [37]	Hungary	Cross-sectional	ELISA IgG, IFA, VNT	3005
Ndione et al., 2022 [38]	Senegal	Cross-sectional	ELISA IgM, PRNT	4715
Negodenko et al., 2021 [39]	Russia	Cross-sectional	ELISA IgM/IgG	806
Odebisi-Omokanye et al., 2024 [40]	Nigeria	Cross-sectional	ELISA IgM, IgG	200
Orf et al., 2024 [41]	Senegal	Cross-sectional	NGS	228
Rakotomalala et al., 2023 [42]	Madagascar	NR	Luminex IgG	1013
Rusanganwa et al., 2023 [43]	Rwanda	Cross-sectional	RT-PCR	2294
Taskin et al., 2023 [44]	Turkey	Cross-sectional	ELISA IgM/IgG, RT-PCR	416
Tinto et al., 2022 [45]	Burkina Faso	Cross-sectional	ELISA, MNT, RT-PCR	689
Toporkov et al., 2022 [46]	Russia	Cross-sectional	ELISA IgM/IgG	1547
Underwood et al., 2024 [47]	USA	Cross-sectional	ELISA IgG, PRNT	250

ELISA – enzyme-linked immunosorbent assay, FRNT – focus reduction neutralisation tests, IgG – immunoglobulin G; IgM – immunoglobulin M, MNT – microneutralisation test, NGS – next-generation sequencing, NR – not reported, PRNT – plaque reduction neutralisation test, RT-PCR – reverse transcription polymerase chain reaction, SNT – serum neutralisation test, VNT – virus neutralisation test, WNV – West Nile virus

*The study included both molecular and serological data on prevalence.

### Pooled global seroprevalence of human WNV

**Figure 2:**
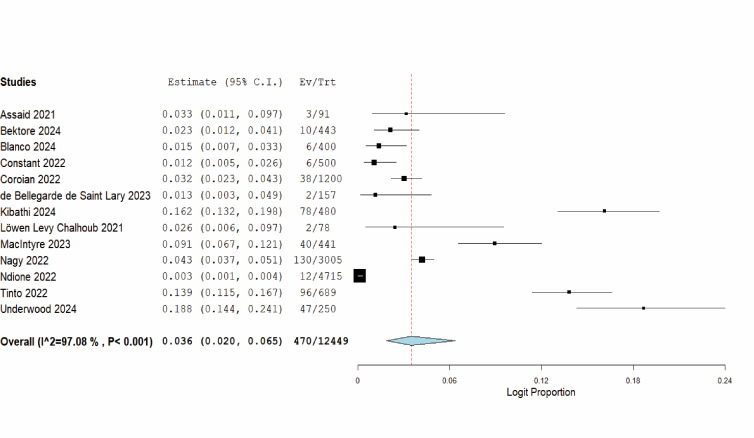
Forest plot of the pooled global seroprevalence of human WNV. WNV – West Nile virus

Given the potential cross-reactivity among flaviviruses, we only included the studies that conducted a confirmatory serological test using a neutralisation assay in the meta-analysis to estimate global seroprevalence. From the 13 studies that met the criteria, we calculated a pooled seroprevalence of 3.6% (95% confidence interval (CI) = 2.0, 6.5). Significant heterogeneity was detected (Q = 441.603; *I*^2^ = 97.08; *P* < 0.001) (**Figure 2**).

**Figure 3:**
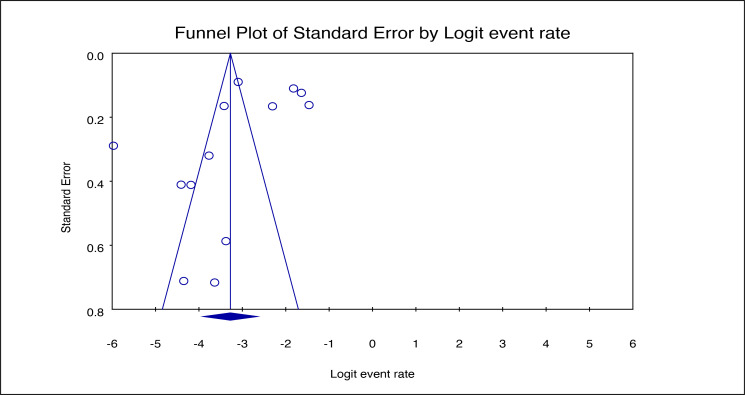
Funnel plot of the studies on global seroprevalence of human WNV (Egger’s *P* = 0.1382). WNV – West Nile virus.

We conducted a sensitivity test using the leave-one-out analysis to assess the stability of the pooled seroprevalence estimate by systematically excluding each study and recalculating the overall estimate. The global seroprevalence of human WNV was stable, with values ranging from 3.1% when removing studies by Kibathi and colleagues [32] and Underwood and colleagues [47], to 4.7% when excluding the study by Ndione and colleagues [38] (Figure S1 in the **Online Supplementary Document**). Further, we generated a funnel plot to evaluate publication bias among the studies contributing to the global seroprevalence estimate. While a visual inspection of the plot indicated potential publication bias, the Egger’s regression test confirmed that there was no significant bias (*P* = 0.1382) (**Figure 3**).

### Reports of human WNV seroprevalence without confirmation

In the meta-analysis, we excluded some studies that met the inclusion criteria because they lacked confirmatory serological testing. These studies were conducted in various regions worldwide, including Russia, Turkey, Madagascar, Nigeria, Australia, Romania, and Palestine. The reported seroprevalence rates varied, ranging from 0.7% in Turkey to 52% in Nigeria (Figure S2, Panel A in the **Online Supplementary Document**). To better understand the potential impact of excluding such studies, we conducted a separate pooled analysis of their reported prevalence. The resulting estimate was 10.9% (95% CI = 5.6, 20.1; Q = 401.010; *I*^2^ = 98.25; *P* < 0.001) (Figure S2, Panel B in the **Online Supplementary Document**), notably higher than the main pooled estimate of 3.6%. This suggests that non-confirmatory serological testing may lead to inflated prevalence estimates, underscoring the importance of confirmatory testing in generating accurate epidemiological data.

### Pooled global molecular prevalence of human WNV

The prevalence of human WNV was evaluated in various studies using molecular techniques like reverse transcription polymerase chain reaction and next-generation sequencing. From the data, the pooled molecular prevalence was 0.1% (95% CI = 0.00, 0.02). Heterogeneity was observed (Q = 35.302; *I*^2^ = 88.67; *P* < 0.001) (Figure S3 in the **Online Supplementary Document**). A leave-one-out sensitivity analysis revealed that the estimates remained stable, ranging from 0% when excluding the Orf and colleagues study [41] to 0.3% when excluding the Kwon and colleagues study [33] (Figure S4 in the **Online Supplementary Document**).

### Subgroup and meta-regression analysis of the global seroprevalence of human WNV

We performed a subgroup meta-analysis to explore regional estimates of human WNV seroprevalence and investigate the significant heterogeneity observed. Although studies contributing to the prevalence data in this work were from various countries worldwide (Figure S5 in the **Online Supplementary Document**), data for regional seroprevalence analysis were available for four regions (**Table 2**). North America had the highest seroprevalence estimate (18.8%), based on a single study, followed by Africa (4.8%), with South America having the lowest (1.7%). Overall, heterogeneity was high in the subgroups, except for studies contributing to the South American estimate, where no heterogeneity was detected (Figure S6 in the **Online Supplementary Document**). Furthermore, except for North America, which showed a significantly higher seroprevalence rate compared to Europe, meta-regression analysis showed that the study region did not significantly influence the pooled seroprevalence estimates (**Table 3**; Figure S7 in the **Online Supplementary Document**).

**Table 2:** Seroprevalence of human WNV in different subgroups

	Number of studies	Prevalence, % (95% CI)	Heterogeneity test		
			**Q**	***I* ^2^**	***P*-value**
**Region**					
Africa	5	4.8 (1.7, 13.2)	205.817	98.06	<0.001
Europe	5	2.6 (1.7, 4.0)	17.000	76.47	0.002
South America	2	1.7 (0.9, 3.4)	0.438	0	0.508
North America	1	18.8 (14.4, 24.1)	NA	NA	NA
**Study population**					
Clinical patients	6	3.2 (1.0, 10.4)	247.764	97.98	<0.001
Blood donors	4	4.7 (1.7, 12.6)	174.227	98.28	<0.001
High-risk individuals	4	2.3 (1.2, 4.4)	1.135	0	0.769

CI – confidence interval, NA – not applicable, WNV – West Nile virus, Q – Cochran’s Q

**Table 3:** Meta-regression analysis of factors affecting heterogeneity

	Coefficient (95% CI)*	*P*-value
**Region**		
Europe	ref	
Africa	0.787 (−0.496, 2.070)	0.229
South America	−0.197 (−2.013, 1.620)	0.832
North America	2.288 (0.135, 4.440)	0.037
**Study population**		
Clinical patients	ref	
Blood donors	0.339 (−1.283, 1.962)	0.682
High-risk individuals	−0.424 (−2.165, 1.317)	0.633

CI – confidence interval, ref – reference *Coefficients quantify how much the average effect size differs between groups, relative to the chosen reference category.

We further stratified the studies into three primary groups based on the characteristics of the study population: clinical patients (including both febrile and afebrile individuals identified through hospital-based surveys or those suspected of WNV infection), blood donors (defined as healthy individuals donating blood), and high-risk individuals (those considered at elevated risk of WNV exposure due to their occupation or environmental conditions). The highest seroprevalence of WNV was observed among blood donors (4.7%), followed by clinical patients (3.2%), and then high-risk individuals (2.3%) (**Table 2**). Heterogeneity was present within the subgroups, except for the high-risk group, where no heterogeneity was observed (Figures S8–10 in the **Online Supplementary Document**). Lastly, meta-regression analysis showed no significant influence of study population on the seroprevalence estimates (**Table 3**; Figure S11 in the **Online Supplementary Document**).

## DISCUSSION

Disease prevalence reports continue to evolve due to changes in surveillance, awareness of prevention and control measures, reporting rates, and advancements in pathogen detection methods. We combined recent global reports on human WNV to provide an overview of the current state of human WNV infections. The results showed a global seroprevalence of 3.6% (95% CI = 2.0, 6.5). Although the seroprevalence we observed appears to be lower compared to estimates from related flaviviruses, this outcome reflects our specific approach to pooled seroprevalence analysis. We only included confirmed cases of WNV infection, verified through neutralisation tests, due to concerns over cross-reactivity between flaviviruses. A prior meta-analysis reported global seroprevalences of 38% for dengue and 18% for Zika infections [48], though these figures may not solely represent confirmed cases. Moreover, we found that human WNV seroprevalence, in the absence of confirmatory testing, ranged widely from 0.7% to 52% depending on the study location, resulting in an inflated pooled estimate of 10.9%. (Figure S2, Panels A and B in the **Online Supplementary Document**). Therefore, we focussed our seroprevalence analysis exclusively on studies that utilised confirmatory diagnostic tests.

We observed the highest seroprevalence in North America (18.8%), although this estimate was based on a single study, followed by Africa at 4.8%. These findings highlight regional differences in WNV exposure, which may be influenced by factors such as vector populations, climate, and the extent of surveillance efforts [49,50]. The higher seroprevalence in North America could be linked to the significant WNV outbreaks that have occurred in recent years, particularly in the USA. The virus has maintained a stable presence in the region since its introduction in 1999, and several large-scale human outbreaks have been documented, resulting in increased awareness and diagnostic testing [51,52]. The study contributing to the elevated North American estimate was a hospital-based investigation conducted in Florida, a region not typically recognised as a major WNV hotspot. Nonetheless, the findings suggest a substantial level of prior exposure among individuals in the area, resulting in a comparatively higher seroprevalence. In contrast, the 4.8% seroprevalence in Africa is relatively lower, despite the virus's widespread presence in some parts of the continent. This may reflect differences in the intensity of WNV surveillance, health care infrastructure, and the capacity for serological testing. Additionally, factors such as mosquito species diversity and environmental conditions may contribute to varied transmission dynamics across regions [50,53].

Of the four regions for which confirmed seroprevalence data were available, we observed that South America had the lowest seroprevalence of human WNV, with an estimate of just 1.7%. While WNV has been detected in several South American countries, large-scale outbreaks have been rare [54]. In 2004, WNV activity was first reported in northern Colombia and Trinidad, marking the initial detection of the virus in South America. By 2006, Argentina also reported evidence of WNV transmission [55]. However, the virus has not yet led to the same scale of human infections observed in North America or Africa. This relatively lower transmission intensity may be influenced by several regional factors, such as less favourable environmental conditions for the primary mosquito vectors (*e.g. Culex* species). Cross-protection from other circulating flaviviruses, such as dengue or Zika, could also contribute to reduced susceptibility [56]. Additionally, surveillance and reporting practices may also contribute to the lower observed seroprevalence. Many South American countries may have less robust WNV surveillance infrastructure compared to North America, where continuous public health monitoring has been in place since the virus was first introduced.

Many of the records included in this analysis originated from middle- and high-income countries, with low-income countries (LICs) being notably underrepresented. The underrepresentation of LICs can be largely attributed to disparities in laboratory capacity and disease surveillance infrastructure. While neutralisation tests are required for definitive WNV confirmation, most low-income settings lack the specialised laboratories, equipment, and trained staff needed to perform these sophisticated assays [57,58]. As a result, LICs often rely on less specific screening methods, such as ELISA, or lack testing altogether. Additionally, weak surveillance systems and limited research funding (which are typically directed toward diseases with higher local burden) may further constrain WNV data generation and reporting. These challenges contribute to the scarcity of peer-reviewed WNV studies from LICs, which in turn affects their representation in meta-analysis and may bias global pooled prevalence estimates.

The subgroup analyses also reveal important insights into population-level risk and exposure patterns. Notably, we found the highest seroprevalence among blood donors (4.7%), followed by clinical patients (3.2%), and high-risk individuals (2.3%). These findings challenge common assumptions about exposure dynamics and may have implications for surveillance and public health prioritisation. The unexpectedly high seroprevalence among blood donors may reflect asymptomatic or subclinical infections, which comprise the majority of WNV cases [14]. Blood donors are typically healthy adults, often prescreened for illness, yet their inclusion in surveillance systems has proven effective in detecting silent circulation of WNV in both endemic and emerging areas [59]. This underscores the importance of blood donation screening using neutralisation assay and nucleic acid testing as a sentinel surveillance tool for reliable and early WNV detection.

Although it was unexpected that high-risk individuals exhibited the lowest WNV seroprevalence, some plausible explanations may account for this finding. People in this group may adopt preventive behaviours, including the use of repellents or protective clothing, especially in occupational settings. While geographic variations in mosquito vector density and virus circulation may influence exposure risk in high-risk occupational settings [60–62], the available data were insufficient to allow a robust assessment of these ecological factors. Additionally, the pooled seroprevalence estimate was derived from studies conducted across diverse geographic regions, which may have masked localised patterns of exposure.

We expected high heterogeneity due to potential variations in several factors, including geographic differences, surveillance intensity, diagnostic methods, and population characteristics. This justified our use of a random-effects meta-analysis model. To investigate potential sources of heterogeneity, we conducted subgroup and meta-regression analyses based on the two covariates (*i.e.* geographic region and study population) for which sufficient data were available. These analyses showed that neither factor significantly explained the heterogeneity, and prevalence estimates were generally consistent across subgroups, except for North America, where prevalence was notably higher than in Europe. However, sensitivity analysis using the leave-one-out method confirmed that the North American study did not excessively influence the overall pooled estimate. Therefore, the substantial heterogeneity may be attributed to other unmeasured factors for which data were unavailable. Although methodological variation is a common contributor to heterogeneity, we minimised this by restricting the analysis to studies employing confirmatory neutralisation testing. Despite the observed high heterogeneity, the pooled prevalence estimate remains robust, supported by relatively narrow CIs.

The estimated pooled molecular prevalence of WNV infection is 0.1%, which may be attributed to the limitations of testing. This low molecular prevalence contrasts with the higher serological prevalence, as molecular diagnostics identify active infections rather than past exposure. Moreover, molecular screening of asymptomatic cases, which characterise most WNV infections, is not a common practice.

As challenges such as asymptomatic infections, climate change, and urbanisation continue to influence and blur the traditional geographic boundaries of WNV transmission, a proactive and globally coordinated response is essential. To address the silent and potentially underestimated burden of WNV, especially in low-prevalence or undersurveillance settings, we propose several implementation strategies. First, WNV screening should be integrated into blood safety protocols, particularly in endemic or high-risk zones, to identify asymptomatic cases and prevent transfusion-related transmission. Second, international investment is needed to strengthen laboratory infrastructure, especially for conducting neutralisation assays, through regional reference laboratories that can support countries lacking local capacity. Third, sentinel surveillance systems should be established using groups such as outdoor workers and residents of mosquito-prone areas, to provide early warning of WNV circulation. Fourth, WNV should be incorporated into climate-sensitive disease monitoring frameworks to anticipate future outbreaks in regions facing ecological shifts favourable to vector expansion. Finally, enhanced cross-border coordination and data sharing are essential, particularly along migratory bird flyways and human migration corridors, to ensure timely detection and containment of transboundary outbreaks.

### Strengths and limitations

We provide a comprehensive synthesis of recent global WNV seroprevalence and molecular prevalence, incorporating data from 24 studies spanning various regions worldwide. By combining recent studies from diverse geographical locations, we have gained a comprehensive understanding of the current status of the virus in humans, providing a robust foundation for the global epidemiology of the virus. However, the analysis was constrained by the inadequate or lack of availability of studies employing confirmatory WNV tests for seroprevalence determination, particularly from Asia and the USA. This disparity likely reflects prioritisation of research funding toward diseases with more pressing local public health impacts, potentially at the expense of comprehensive WNV surveillance. Consequently, limited diagnostic efforts and underrepresentation of certain regions may reduce the global generalisability and precision of our findings. For example, we derived the estimate for North America from a single hospital-based study conducted in Tampa Bay, Florida, which may not be representative of the broader national prevalence and should thus be interpreted with caution. These limitations underscore the urgent need for expanded surveillance and seroepidemiological research in underrepresented regions to generate more accurate and comprehensive global estimates. Nonetheless, despite these constraints, our study serves as a valuable resource for informing public health strategies to address the impact of WNV infection.

## CONCLUSIONS

This meta-analysis offers a comprehensive overview of the current global WNV seroprevalence and molecular prevalence in the human population, highlighting considerable regional variability and emphasising the necessity for tailored public health interventions. Although the overall global prevalence appears low, this may be due to underreporting and limited diagnostic capacity, particularly in low-resource settings. Our findings underscore the importance of improved WNV surveillance efforts worldwide, especially in underrepresented regions. To better capture the true burden of WNV and guide effective responses, there is an urgent need to scale up integrated surveillance efforts. These should include routine prevalence monitoring within public health systems, targeted blood donor screening in endemic and high-risk regions, and the establishment of sentinel surveillance programmes in birds, mosquitoes, and high-risk human populations. Standardised diagnostic protocols, particularly the expanded use of confirmatory neutralisation assays in seroprevalence assessment, should be developed and implemented globally to enhance data reliability and comparability. Considering shifting ecological and demographic factors, longitudinal studies are needed to track the evolving epidemiology of WNV and to inform timely, data-driven public health responses. Strengthening global coordination, laboratory capacity, and early warning systems will be critical to mitigating the silent but potentially expanding threat of WNV as a global public health concern.

## Additional material


Online Supplementary Document

## References

[1] Dauphin G Zientara S Zeller H Murgue B West Nile: worldwide current situation in animals and humans. Comp Immunol Microbiol Infect Dis. 2004;27:343–55. 10.1016/j.cimid.2004.03.009 15225984

[2] Klingelhöfer D Braun M Kramer IM Reuss F Müller R Groneberg DA A virus becomes a global concern: research activities on West-Nile virus. Emerg Microbes Infect. 2023;12:2256424. 10.1080/22221751.2023.2256424 37671854 PMC10501173

[3] Athni TS Shocket MS Couper LI Nova N Caldwell IR Caldwell JM The influence of vector-borne disease on human history: socio-ecological mechanisms. Ecol Lett. 2021;24:829–46. 10.1111/ele.13675 33501751 PMC7969392

[4] Simonin Y Circulation of West Nile Virus and Usutu Virus in Europe: Overview and Challenges. Viruses. 2024;16:599. 10.3390/v16040599 38675940 PMC11055060

[5] Colpitts TM Conway MJ Montgomery RR Fikrig E West Nile Virus: Biology, Transmission, and Human Infection. Clin Microbiol Rev. 2012;25:635–48. 10.1128/CMR.00045-12 23034323 PMC3485754

[6] Singh P Khatib MN Ballal S Kaur M Nathiya D Sharma S West Nile Virus in a changing climate: epidemiology, pathology, advances in diagnosis and treatment, vaccine designing and control strategies, emerging public health challenges – a comprehensive review. Emerg Microbes Infect. 2025;14:2437244. 10.1098/rstb.2013.0561 39614679 PMC11703391

[7] Sule WF Oluwayelu DO Hernández-Triana LM Fooks AR Venter M Johnson N Epidemiology and ecology of West Nile virus in sub-Saharan Africa. Parasit Vectors. 2018;11:414. 10.1186/s13071-018-2998-y 30005653 PMC6043977

[8] Habarugira G Suen WW Hobson-Peters J Hall RA Bielefeldt-Ohmann H West Nile virus: an update on pathobiology, epidemiology, diagnostics, control and “one health” implications. Pathogens. 2020;9:589. 10.3390/pathogens9070589 32707644 PMC7400489

[9] Bialosuknia SM Dupuis Ii AP Zink SD Koetzner CA Maffei JG Owen JC Adaptive evolution of West Nile virus facilitated increased transmissibility and prevalence in New York State. Emerg Microbes Infect. 2022;11:988–99. 10.1080/22221751.2022.2056521 35317702 PMC8982463

[10] Neiderud CJ How urbanization affects the epidemiology of emerging infectious diseases. Infect Ecol Epidemiol. 2015;5:27060. 10.3402/iee.v5.27060 26112265 PMC4481042

[11] Paz S Climate change impacts on West Nile virus transmission in a global context. Philos Trans R Soc Lond B Biol Sci. 2015;370:20130561. 10.1098/rstb.2013.0561 25688020 PMC4342965

[12] Marlina S Radzi SFM Lani R Sieng KC Rahim NFA Hassan H Seroprevalence screening for the West Nile virus in Malaysia’s Orang Asli population. Parasit Vectors. 2014;7:597. 10.1186/s13071-014-0597-0 25515627 PMC4311511

[13] Marra PP Griffing S Caffrey C Kilpatrick MA McLean R Brand C West Nile virus and wildlife. Bioscience. 2024;54:393–402. 10.1016/j.onehlt.2022.100478 37363246 PMC10288031

[14] Bampali M Konstantinidis K Kellis EE Pouni T Mitroulis I Kottaridi C West Nile disease symptoms and comorbidities: a systematic review and analysis of cases. Trop Med Infect Dis. 2022;7:236. 10.3390/tropicalmed7090236 36136647 PMC9506265

[15] Dias BP Barbosa CC Ferreira CS Mayra Soares Alves Dos Santos S Arrieta OAP Malta WC Challenges in direct detection of flaviviruses: a review. Pathogens. 2023;12:643. 10.3390/pathogens12050643 37242313 PMC10223438

[16] Chan KR Ismail AA Thergarajan G Raju CS Yam HC Rishya M Serological cross-reactivity among common flaviviruses. Front Cell Infect Microbiol. 2022;12:975398. 10.3389/fcimb.2022.975398 36189346 PMC9519894

[17] Sejvar JJ West Nile Virus Infection. Microbiol Spectr. 2016;4. 10.1128/microbiolspec.EI10-0021-2016 27337465

[18] Moher D Liberati A Tetzlaff J Altman DG Altman D Antes G Preferred reporting items for systematic reviews and meta-analyses: the PRISMA statement. Ann Intern Med. 2009;151:264–9. 10.7326/0003-4819-151-4-200908180-00135 19622511

[19] Egger M Davey Smith G Schneider M Minder C Bias in meta-analysis detected by a simple, graphical test. BMJ. 1997;315:629–34. 10.1136/bmj.315.7109.629 9310563 PMC2127453

[20] Higgins JPT Thompson SG Quantifying heterogeneity in a meta-analysis. Stat Med. 2002;21:1539–58. 10.1002/sim.1186 12111919

[21] Alshehri AA Irekeola AA Global prevalence of alkhumra hemorrhagic fever virus infection: The first meta-analysis and systematic review. J Infect Public Health. 2024;17:986–93. 10.1016/j.jiph.2024.04.001 38631068

[22] Munn Z Moola S Lisy K Riitano D Tufanaru C Methodological guidance for systematic reviews of observational epidemiological studies reporting prevalence and cumulative incidence data. Int J Evid-Based Healthc. 2015;13:147–53. 10.1097/XEB.0000000000000054 26317388

[23] Irekeola AA Engku Nur Syafirah EAR Islam MA Shueb RH Global prevalence of dengue and chikungunya coinfection: A systematic review and meta-analysis of 43,341 participants. Acta Trop. 2022;231:106408. 10.1016/j.actatropica.2022.106408 35305942

[24] Alzuheir I Fayyad A Jalboush N Abdallah R Abutarbush S Gharaibeh M Seroprevalence and risk factors of West Nile virus infection in veterinarians and horses in Northern Palestine. Vet World. 2021;14:1241–6. 10.14202/vetworld.2021.1241-1246 34220126 PMC8243691

[25] Assaid N Arich S Ezzikouri S Benjelloun S Dia M Faye O Serological evidence of West Nile virus infection in human populations and domestic birds in the Northwest of Morocco. Comp Immunol Microbiol Infect Dis. 2021;76:101646. 10.1016/j.cimid.2021.101646 33845402

[26] Bektore B Dogan B Ozkul A Gozalan A West Nile virus seropositivity in Alanya, a coastal city in the Mediterranean region of Turkey. Ann Saudi Med. 2024;44:48–54. 10.5144/0256-4947.2024.48 38311862 PMC10839453

[27] Blanco S Marín ÁL Frutos MC Barahona NY Rivarola ME Carrizo LH Haemovigilance survey and screening strategy for arthropod-borne viruses in blood donors from Argentina. J Med Virol. 2024;96:e29476. 10.1002/jmv.29476 38373210

[28] Constant O Gil P Barthelemy J Bolloré K Foulongne V Desmetz C One Health surveillance of West Nile and Usutu viruses: a repeated cross-sectional study exploring seroprevalence and endemicity in Southern France, 2016 to 2020. Euro Surveill. 2022;27:2200068. 10.2807/1560-7917.ES.2022.27.25.2200068 35748300 PMC9229194

[29] Coroian M Mihalca AD Dobler G Euringer K Girl P Borșan SD Seroprevalence Rates against West Nile, Usutu, and Tick-Borne Encephalitis Viruses in Blood-Donors from North-Western Romania. Int J Environ Res Public Health. 2022;19:8182. 10.3390/ijerph19138182 35805850 PMC9266370

[30] Crivei LA Vata A Teodor D Porea D Cozma AP Anita A An Assessment of West Nile and Usutu Viruses’ Seroprevalence in Hospitalized Patients: A Preliminary Study on Flavivirus Exposure in Eastern Romania. Pathogens. 2024;13:133. 10.3390/pathogens13020133 38392871 PMC10892458

[31] de Bellegarde de Saint Lary C Kasbergen LMR Bruijning-Verhagen PPCJL van der Jeugd H Chandler F Hogema BM Assessing West Nile virus (WNV) and Usutu virus (USUV) exposure in bird ringers in the Netherlands: a high-risk group for WNV and USUV infection? One Health. 2023;16:100533. 10.1016/j.onehlt.2023.100533 37363259 PMC10288042

[32] Kibathi MH Chepkorir E Mabeya SN Tchouassi DP Sang R Seroprevalence of dengue, yellow fever, and related flaviviruses among the rural human population in Nguruman and Kerio Valley, Kenya. Front Virol. 2024;4:1459021. 10.3389/fviro.2024.1459021 PMC12998444 41859757

[33] Kwon S Casleton BG Rivera GZ Gella MM Winkler EL Kieffer JW Infectious etiologies among post-donation deferrals in a military blood donation center. Transfusion. 2023;63:2265–72. 10.1111/trf.17584 37850496

[34] Löwen Levy Chalhoub F Maia de Queiroz-Júnior E Holanda Duarte B Eielson Pinheiro de Sá M Cerqueira Lima P Carneiro de Oliveira A West Nile Virus in the State of Ceará, Northeast Brazil. Microorganisms. 2021;9:1699. 10.3390/microorganisms9081699 34442778 PMC8401605

[35] MacIntyre C Lourens C Mendes A de Villiers M Avenant T du Plessis NM West Nile Virus, an Underdiagnosed Cause of Acute Fever of Unknown Origin and Neurological Disease among Hospitalized Patients in South Africa. Viruses. 2023;15:2207. 10.3390/v15112207 38005884 PMC10674603

[36] Marsland MJ Thomson TN O’Brien HM Peach E Bellette J Humphreys N Serosurvey for Japanese encephalitis virus antibodies following an outbreak in an immunologically naïve population, Victoria, 2022: a cross-sectional study. Med J Aust. 2024;220:566–72. 10.5694/mja2.52344 38803004

[37] Nagy A Csonka N Takács M Mezei E Barabás É West Nile and Usutu virus seroprevalence in Hungary: A nationwide serosurvey among blood donors in 2019. PLoS One. 2022;17:e0266840. 10.1371/journal.pone.0266840 35395048 PMC8992992

[38] Ndione MHD Ndiaye E Faye M Diagne MM Diallo D Diallo A Re-Introduction of West Nile Virus Lineage 1 in Senegal from Europe and Subsequent Circulation in Human and Mosquito Populations between 2012 and 2021. Viruses. 2022;14:2720. 10.3390/v14122720 36560724 PMC9785585

[39] Negodenko AO Molchanova EV Prilepskaya DR Konovalov P Pavlyukova OA Skrynnikova EA [Analysis of the results of monitoring arbovirus infections in the Volgograd region in 2019]. Epidemiology and Vaccinal Prevention. 2021;20:51–9. Russian. 10.31631/2073-3046-2021-20-1-51-59

[40] Odebisi-Omokanye MB Suleiman MM Sulaiman MK Atolagbe SA Seropositivity of West Nile virus among acute febrile patients in Ilorin, Nigeria. Vopr Virusol. 2024;69:320–8. 10.36233/0507-4088-241 39361926

[41] Orf GS Ahouidi AD Mata M Diedhiou C Mboup A Padane A Next-generation sequencing survey of acute febrile illness in Senegal (2020-2022). Front Microbiol. 2024;15:1362714. 10.3389/fmicb.2024.1362714 38655084 PMC11037400

[42] Rakotomalala FA Bouillin J Randriarimanana SD Thaurignac G Maharavo L Raberahona M High Seroprevalence of IgG Antibodies to Multiple Arboviruses in People Living with HIV (PLWHIV) in Madagascar. Viruses. 2023;15:2258. 10.3390/v15112258 38005934 PMC10674502

[43] Rusanganwa V Lwande OW Bainda B Chiyo PI Seruyange E Bucht G Arbovirus surveillance in febrile patients attending selected health facilities in Rwanda. Infect Ecol Epidemiol. 2023;14:2289872. 40181819 10.1080/20008686.2023.2289872 PMC11967279

[44] Taskin MH Tamer C Muftuoglu B Ozan E Kilic SS Akkoyunlu GK The first serological detection of West Nile virus infection among residents living in northern Turkey. J Vector Borne Dis. 2023;60:101–5. 10.4103/0972-9062.364755 37026226

[45] Tinto B Kaboré DPA Kagoné TS Constant O Barthelemy J Kiba-Koumaré A Screening of Circulation of Usutu and West Nile Viruses: A One Health Approach in Humans, Domestic Animals and Mosquitoes in Burkina Faso, West Africa. Microorganisms. 2022;10:2016. 10.3390/microorganisms10102016 36296292 PMC9610586

[46] Toporkov AV Putintseva EV Udovichenko SK Boroday NV Molchanova EV Bondareva OS Study of the circulation and properties of the West Nile virus in Russia in 2022. Journal of Microbiology, Epidemiology and Immunobiology. 2024;101:114–26. 10.36233/0372-9311-432

[47] Underwood EC Vera IM Allen D Alvior J O’Driscoll M Silbert S Seroprevalence of West Nile Virus in Tampa Bay Florida Patients Admitted to Hospital during 2020-2021 for Respiratory Symptoms. Viruses. 2024;16:719. 10.3390/v16050719 38793601 PMC11125834

[48] Li Z Wang J Cheng X Hu H Guo C Huang J The worldwide seroprevalence of DENV, CHIKV and ZIKV infection: A systematic review and meta-analysis. PLoS Negl Trop Dis. 2021;15:e0009337. 10.1371/journal.pntd.0009337 33909610 PMC8109817

[49] McDonald E Mathis S Martin SW Staples JE Fischer M Lindsey NP Surveillance for West Nile virus disease—United States, 2009–2018. MMWR Surveill Summ. 2021;70:1–15. 10.15585/mmwr.ss7001a1 33661868 PMC7949089

[50] Giesen C Herrador Z Fernandez-Martinez B Figuerola J Gangoso L Vazquez A A systematic review of environmental factors related to WNV circulation in European and Mediterranean countries. One Health. 2023;16:100478. 10.1016/j.onehlt.2022.100478 37363246 PMC10288031

[51] Petersen LR Brault AC Nasci RS West Nile Virus: Review of the Literature. JAMA. 2013;310:308–15. 10.1001/jama.2013.8042 23860989 PMC4563989

[52] Sejvar JJ West Nile virus: an historical overview. Ochsner J. 2003;5:6–10. 21765761 PMC3111838

[53] Luethy D Eastern, Western, and Venezuelan Equine Encephalitis and West Nile Viruses: Clinical and Public Health Considerations. Vet Clin North Am Equine Pract. 2023;39:99–113. 10.1016/j.cveq.2022.11.007 36737290

[54] Lorenz C Chiaravalloti-Neto F Why are there no human West Nile virus outbreaks in South America? Lancet Reg Health Am. 2022;12:100276. 10.1016/j.lana.2022.100276 36776433 PMC9903813

[55] Gubler DJ The Continuing Spread of West Nile Virus in the Western Hemisphere. Clin Infect Dis. 2007;45:1039–46. 10.1086/521911 17879923

[56] Rathore APS St. John AL Cross-Reactive Immunity Among Flaviviruses. Front Immunol. 2020;11:334. 10.3389/fimmu.2020.00334 32174923 PMC7054434

[57] Nkengasong JN Yao K Onyebujoh P Laboratory medicine in low-income and middle-income countries: progress and challenges. Lancet. 2018;391:1873–5. 10.1016/S0140-6736(18)30308-8 29550031 PMC5962422

[58] Yadav H Shah D Sayed S Horton S Schroeder LF Availability of essential diagnostics in ten low-income and middle-income countries: results from national health facility surveys. Lancet Glob Health. 2021;9:e1553–60. 10.1016/S2214-109X(21)00442-3 34626546 PMC8526361

[59] Glynn SA Busch MP Dodd RY Katz LM Stramer SL Klein HG Emerging infectious agents and the nation’s blood supply: responding to potential threats in the 21st century. Transfusion. 2013;53:438–54. 10.1111/j.1537-2995.2012.03742.x 22690676 PMC3644861

[60] Bisanzio D Giacobini M Bertolotti L Mosca A Balbo L Kitron U Spatio-temporal patterns of distribution of West Nile virus vectors in eastern Piedmont Region, Italy. Parasit Vectors. 2011;4:230. 10.1186/1756-3305-4-230 22152822 PMC3251540

[61] Carnes A Ogneva-Himmelberger Y Temporal variations in the distribution of West Nile virus within the United States; 2000–2008. Appl Spat Anal Policy. 2012;5:211–29. 10.1007/s12061-011-9067-7 32218878 PMC7090722

[62] Durand B Tran A Balança G Chevalier V Geographic variations of the bird-borne structural risk of West Nile virus circulation in Europe. PLoS One. 2017;12:e0185962. 10.1371/journal.pone.0185962 29023472 PMC5638290

